# Current and Future Intraoperative Imaging Strategies to Increase Radical Resection Rates in Pancreatic Cancer Surgery

**DOI:** 10.1155/2014/890230

**Published:** 2014-07-15

**Authors:** Henricus J. M. Handgraaf, Martin C. Boonstra, Arian R. Van Erkel, Bert A. Bonsing, Hein Putter, Cornelis J. H. Van De Velde, Alexander L. Vahrmeijer, J. Sven D. Mieog

**Affiliations:** ^1^ Department of Surgery, Leiden University Medical Center, Albinusdreef 2, 2300 RC Leiden, The Netherlands; ^2^Department of Radiology, Leiden University Medical Center, Albinusdreef 2, 2300 RC Leiden, The Netherlands; ^3^Department of Medical Statistics, Leiden University Medical Center, Albinusdreef 2, 2300 RC Leiden, The Netherlands

## Abstract

Prognosis of patients with pancreatic cancer is poor. Even the small minority that undergoes resection with curative intent has low 5-year survival rates. This may partly be explained by the high number of irradical resections, which results in local recurrence and impaired overall survival. Currently, ultrasonography is used during surgery for resectability assessment and frozen-section analysis is used for assessment of resection margins in order to decrease the number of irradical resections. The introduction of minimal invasive techniques in pancreatic surgery has deprived surgeons from direct tactile information. To improve intraoperative assessment of pancreatic tumor extension, enhanced or novel intraoperative imaging technologies accurately visualizing and delineating cancer cells are necessary. Emerging modalities are intraoperative near-infrared fluorescence imaging and freehand nuclear imaging using tumor-specific targeted contrast agents. In this review, we performed a meta-analysis of the literature on laparoscopic ultrasonography and we summarized and discussed current and future intraoperative imaging modalities and their potential for improved tumor demarcation during pancreatic surgery.

## 1. Introduction

Surgery is the cornerstone of curative intended treatment of pancreatic cancer [1]. However, resection of pancreatic cancer is only suitable for a minority of patients [2, 3]. Pancreatic cancer surgery is only conducted when there is a reasonable chance of complete removal of all cancer cells (radical resection), as irradical resection does not improve survival but elicit procedure-related morbidity and mortality [4]. Consequently, pancreatic cancer is known for its high mortality and low 5-year survival of only 6% [5].

Despite recent advances in preoperative imaging modalities, such as computed tomography (CT) and magnetic resonance imaging (MRI), the preoperative assessment of resectability is limited due to difficult differentiation of necrosis, fibrosis, and edematous tissue from malignant tumor cells, especially after neoadjuvant therapy [6–9]. The combination of endoscopic ultrasonography (EUS) and laparoscopic ultrasonography improves resectability assessment [10, 11]. However, microscopic involvement of resection margins (R1 resection) is reported up to 75% of cases, which results in local recurrences and decreased overall survival [12–17]. Therefore, intraoperative imaging strategies accurately visualizing pancreatic cancer cells are highly necessary.

Preoperative imaging of pancreatic cancer using CT, MRI, single-photon emission CT (SPECT), positron emission tomography (PET), and EUS enhances surgical planning, but translating these results to the operating room is difficult due to altered body positioning, tissue manipulation by the surgeon, and lack of sensitivity for subcentimeter lesions. When laparotomy is performed, careful palpation and inspection can yield more information about tumor localization. Minimal-invasive techniques have become important in daily clinical practice but limit tactile feedback.

**Figure 1:**
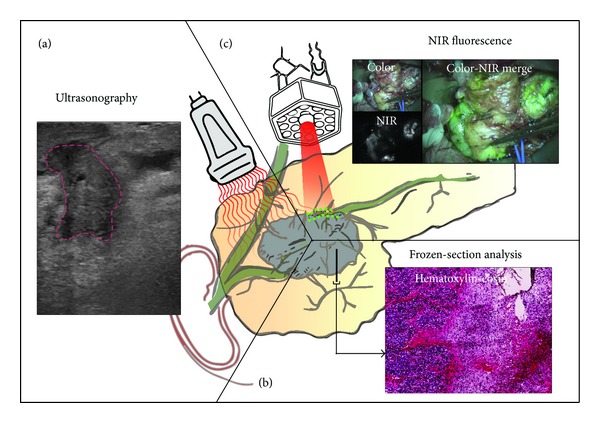
Schematic overview of current and future intraoperative imaging modalities. (a) Ultrasonography showing a pancreatic tumor (demarcated with red line). (b) Intraoperative frozen section analysis and (c) optical imaging using near-infrared imaging.

In conclusion, there is a dire need for imaging techniques accurately visualizing and delineating pancreatic cancer during surgery. This review discusses current techniques that are used to assess pancreatic tumor extension during surgery and evaluates the most promising future imaging techniques (Figure 1).

## 2. Current Strategies

### 2.1. Ultrasonography during Surgery

Ultrasonography (US) is a safe and inexpensive modality that can be used for determination of resectability and identification of metastases (Figure 1(a)) [18–21]. Besides percutaneous application, US is also used during laparoscopy (laparoscopic ultrasonography, LUS) and open surgery (intraoperative ultrasonography, IOUS). Compared to palpation and visual inspection, US is less sensitive for surface evaluations but outperforms in examination of the interior of organs and helps to determine blood flow in vasculature [22]. Its user dependency is a limitation; substantial training and experience are required for generating and interpreting useful images during pancreatic cancer surgery. Furthermore, ultrasound waves are unable to penetrate through air or gas, hampering the visibility of structures and organs located behind hollow organs. But, by slight compression or by imaging from another side, this limitation can mostly be overcome.

**Figure 2:**
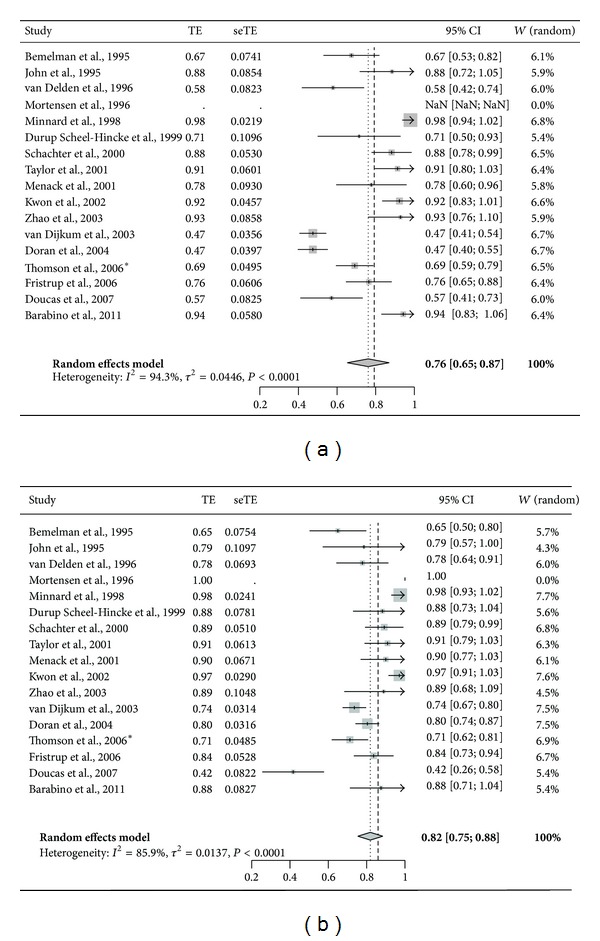
Forest plot of pooled data on (a) sensitivity and (b) negative predictive value of laparoscopic ultrasonography in predicting unresectability of pancreatic cancer, which is preoperatively considered to be resectable. ∗Thomson et al. included 152 patients, 61% had pancreatic adenocarcinoma, 12% presumed pancreatic cancer, 11% ampullary cancer, 5% cholangiocarcinoma, and 11% had other diagnoses. No data solely describing pancreatic cancer patients was available.

Various studies have evaluated the role of LUS in predicting tumor resectability during staging laparoscopy [23–39]. The term “resectability” is used to indicate if radical resection (R_0_) of the tumor is technically possible in the absence of vascular involvement and distance metastases. Staging laparoscopy combined with LUS is not always used to determine resectability, since it is debated whether this approach should be offered routinely, selectively, or not at all to those who appear resectable during their preoperative workup [38, 40]. We performed an extensive review of the literature and pooled the available data in a meta-analysis. We included seventeen studies published between 1995 and 2011. We excluded individual patients from the meta-analysis; if patients were diagnosed with unresectable pancreatic cancer during their preoperative workup, but underwent palliative surgery [27, 29, 34, 36, 38], patients did not undergo LUS, but only laparoscopy [34, 36, 37]; patients declined surgery [31, 37]; if patients were diagnosed with other pathology then pancreatic cancer [31, 32, 35, 37]. In two studies on selective use of LUS, it was not possible to extract the subpopulation of patients that received LUS assessment [41, 42]. Therefore, these studies were not included. In total, data on 1,255 patients undergoing LUS were available for the meta-analysis. A random effect model was chosen due to significant heterogeneity between studies. Pooled sensitivity of LUS for determining unresectable disease was 76% (95% CI = 65–87%) and negative predictive value, the proportion of patients correctly diagnosed with resectable disease, was 82% (95% CI = 75–88%) (Figure 2). The variance between studies may partly be explained by a difference in* a priori *probability, potentially as a result of patient selection by different preoperative imaging modalities. Furthermore, different criteria for unresectability were used.

We were unable to find any study on the use of IOUS in determining resectability, except some outdated literature, which reported success with IOUS in visualizing nonpalpable pancreatic masses [43].

Besides assessing localization and characteristics of the primary tumor, US can also be used for the detection of previously unnoticed metastases. Sensitivity of laparoscopy combined with LUS reached 100% in detecting hepatic and peritoneal metastases in a study of 26 patients with pancreatic cancer compared with percutaneous US, CT, or EUS [44].

US is a useful intraoperative imaging technique and provides valuable information about size, localization, and characteristics of lesions. By intraoperative suspicion of unresectability, LUS can aid in avoiding futile resections, and even more when combined with pretherapeutic EUS [10, 11, 36, 45]. However, little literature exists about the value of US-guided surgery in reducing positive resection margins in pancreatic cancer surgery.

### 2.2. Intraoperative Frozen-Section Analysis

Intraoperative frozen-section analysis (IFSA) of the margins in the pancreatic neck is commonly performed and currently considered as the most important method for intraoperative assessment of the resection margin (Figure 1(b)). It is safe, fast and easy to perform; however, it requires significant processing and evaluating time [46]. Additional resection in case of positive resection margins seems logical, but several studies describe no significant survival benefit after reresection [47–49]. However, no standardized protocol for frozen sections of pancreatic cancer resection margins was described in the studies. The use of nonstandardized methods for histopathological analysis greatly influences the reporting of resection margin status [13, 17, 50]. This may explain the low sensitivity of only 33% in evaluating final resection margin status using IFSA [46]. Due to this inconsistent reporting, little is known on the relation between exact tumor location within the pancreas and margin involvement. When standardized protocols are used, IFSA can potentially be a good method for resection margin assessment. However, IFSA will not provide visual and real-time feedback.

## 3. Future Strategies

### 3.1. Contrast-Enhanced Ultrasonography

Ultrasonography is very usable during pancreatic surgery; hence improvements such as contrast-enhanced US (CEUS) are currently being studied. CEUS uses intravenously administered microbubbles, which allow better determination of vessel infiltration and improved visualization of tumor margins during percutaneous imaging [51–54]. Furthermore, CEUS has already shown to help differentiate between chronic pancreatitis and ductal carcinoma [55]. Finally, CEUS can potentially help in identifying more hepatic metastases [56]. During open resection of colorectal liver metastases, the use of CEUS was of significant value in assessing adequate margins and detecting additional lesions. Preoperative CEUS results are encouraging; translation to the operation room is required to fully study the added value of CEUS during pancreatic cancer surgery.

### 3.2. Fluorescence-Guided Surgery

Fluorescence-guided surgery has emerged as a novel intraoperative modality to assist surgeons to visualize tumors, sentinel lymph nodes, and vital structures in real time (Figure 1(c)) [57]. Near-infrared (NIR) light (700–900 nanometers) can penetrate through several millimeters tissue, revealing targets below the tissue surface [58]. Consequently, NIR fluorescence imaging is currently a surface technique.

At present only two NIR fluorochromes are FDA approved and can be used in the clinical setting, namely, indocyanine green and methylene blue. Both fluorochromes are nonspecific and are mainly used for sentinel lymph node mapping, bile duct imaging, and ureter visualization [57]. Indocyanine green has been shown to accumulate around hepatic metastasis of pancreatic and colorectal cancers, probably due to retention of indocyanine green in compressed hepatocytes, which is shown by a fluorescent rim [59, 60]. In 16% of patients undergoing pancreatic resection without preclinical detected hepatic metastases, fluorescence imaging revealed micrometastases of at least 1.5 mm, which was confirmed by histopathological examination. By revealing undetected hepatic metastases, NIR fluorescence imaging can further decrease the rate of futile pancreatic resections. Furthermore, although its mechanism is unknown, we and others showed that methylene blue tends to accumulate in neuroendocrine tumors, including pancreatic insulinomas [61, 62]. However, due to the nonspecificity no tumor-specific targeting can be expected of ICG and MB, as was shown by our group in pancreatic carcinomas [63]. To obtain the full advantages of NIR fluorescence imaging for pancreatic cancer visualization, tumor specific NIR conjugated ligands need to be designed and tested.

The biological tumor makeup can be used to visualize pancreatic tumors. In the last decades, research on pancreas carcinoma proteomics gained more attention. An increasing number of differentially expressed proteins are identified (http://www.pancreasexpression.org/). Although very promising, these biomarker studies focus mainly on diagnosis or prevention and not necessarily on biomarkers which can be used to recognize malignant cells and to function as tumor-specific target. Potential biomarker for these approaches must possess additional characteristics, such as homogenic expression, upregulation of more than ten times compared to the surrounding tissue, and localization on the cellular membrane for better accessibility. Ideally, these biomarkers can also recognize precursor lesions at early stages and distinguish between pancreatic cancer and inflammation.

Until now, no membrane-bound biomarkers are validated in the clinic, but recent literature shows very promising results in preclinical studies. Various forms of CEA, integrins, BRCA1, and tumor-associated glycoprotein-17 (TAG-17) are overexpressed on pancreatic tumor cells while c-MET, EpCAM, and CXCR-4 are also used as pancreatic cancer stem cell markers [64, 65]. Biomarkers from the plasmin(ogen) cascade are frequently associated with early stage invasion and cell dissociation [66]. The urokinase receptor (uPAR) is highly upregulated on pancreatic tumors and is associated with tumor invasion and its soluble variant differentiates between pancreatic adenocarcinomas and chronic pancreatitis [67, 68].

Very promising preclinical results are already reported for pancreatic cancer specific molecular targets like CEA, MMPs, claudin-4, RGD, and cholecystokinin-2 receptor [69–73]. The focus within the field is currently shifting towards clinical translation and the first successful in-human results of tumor targeted probes have already been published, although this study is concerning ovarian cancer patients [74]. Real-time NIR fluorescence imaging using tumor-targeted probes has the potential to accurately visualize tumor and its demarcation and hence to increase radical resection rates. The next steps should be clinical translation of pancreatic cancer specific probes, improving commercially available NIR fluorescence imaging systems, and validation of the benefits for patients.

### 3.3. Nuclear Imaging

Besides fluorophores, ligands can also be conjugated to radiotracers, which are directed to tumor-specific biomarkers eliciting tumor specific signals and enhancing tumor visualization (Figure 1(d)). These radioactive ligands are used in the preoperative setting with PET and SPECT and intraoperative with radioimmunoguided surgery (RIGS). RIGS was first described in 1984 by Aitken et al., who developed a hand-held gamma detector that can be used intraoperatively, but the technique has become relatively redundant due to the variable sensitivity, the delay in imaging of nearly a week (due to clearance of unbound antibody from the body), and difficulties in handling and disposing the radioactive material [75–78]. A relatively new nuclear imaging technique is freehand SPECT (fSPECT), which was lately introduced as a three-dimensional (3D) imaging and navigation technique that provides real-time images designed for use in the operating room to facilitate detection and resection [79]. However, this technique shows promising results in lymphatic mapping in breast cancer and for the visualization of thyroid diseases but not yet for pancreatic cancer where no known literature exists [79, 80].

## 4. Discussion

The field of pancreatic cancer surgery is changing due to improvements in therapies and imaging modalities. These advances have not only led to better pancreatic cancer surgery but also to limitations. The introduction of laparoscopic techniques, for example, has resulted in less postoperative pain, shorter hospital stay, and lower morbidity [81, 82]. However, laparoscopy deprives the surgeon of tactile information, which is helpful during pancreatic cancer surgery. Another example is neoadjuvant therapy, after which a proportion of patients becomes eligible for curative-intended surgery [83]. However, preoperative imaging modalities, such as CT and MRI, drop in sensitivity and specificity in patients who received chemotherapy, for instance, because they cannot accurately distinguish between vascular involvement or vascular encasement only due to periarterial stranding and fibrosis [6–9].

Intraoperative imaging modalities, which can accurately depict pancreatic cancer, can overcome these limitations. Current available technologies, such as US, have their own limitations. US-guided surgery failed to decrease the rate of R_1_ resections, possibly due to the fact that quality of obtained images is not high enough. The combination of different imaging modalities has proven to be a successful way to overcome separate limitations; PET/CT, for example, fuses anatomical and functional images in a single scan [84]. The combination of US with other techniques could increase functionality. A potential hybrid concept is fSPECT/US, which has already proven to be possible and easy to perform [80].

Molecular imaging is another promising research field where improvements may be expected. NIR fluorescence imaging offers visual guidance during surgery and can therefore potentially reduce the rate of positive resection margins. Fluorescence-guided laparoscopy during hepatopancreatobiliary surgery has already shown its potential to improve intraoperative identification and demarcation of tumors [85]. Remaining fluorescence signal in the resection wound can be an indication of irradical resection, which may make IFSA redundant. To date, no in-human trials have been done with pancreatic cancer specific contrast agents, but preclinical studies are very promising. A major restriction of NIR fluorescence imaging is its limited penetration depth; fluorescence signal is diminished within one centimeter tissue. This is an issue due to the retroperitoneal location of the pancreas. But again, fusing technologies could overcome this limitation. Radiolabeled NIR fluorescence probes may result in the best of two worlds: direct optical guidance and high penetration capacity of the radiotracer [86]. In addition, preoperative planning is possible with PET detection of the radiotracer [87]. Another improvement should be the development of novel probes that are highly specific for pancreatic cancer cells only and hence result in less background signal and lower false-positive rates. Improved fluorophores, which can easily be conjugated to different ligands, are already available, such as ZW800-1 and CW800 [88]. Furthermore, improved imaging systems should become commercially available, making NIR fluorescence imaging available to a broader range of hospitals. But before NIR fluorescence imaging or a hybrid approach can lead to change in patient management, large multicenter studies are necessary to show if these intraoperative imaging modalities are beneficial for patients.

## 5. Conclusion

To improve surgical outcome, reduce irradical resections, and improve patients' survival, novel intraoperative imaging strategies are necessary in pancreatic cancer surgery. Therefore, enhanced imaging technologies that can accurately visualize and delineate pancreatic cancer and its extension in real time are currently being developed and tested. Tumor-specific targeted probes for near-infrared fluorescence imaging are very promising, but research in the next years will have to determine if these modalities are truly of added value for our patients.
